# Chronic *Pseudomonas aeruginosa* Pneumonia Triggers Inflammation-Driven Oncogenic Signaling in Juvenile Mice: Implications for Pharmacological Intervention

**DOI:** 10.5812/ijpr-163368

**Published:** 2025-07-28

**Authors:** Jing Zhang, Zahra Zahid Piracha, Umar Saeed, Dilber Uzun Ozsahin, Muhammad Waseem, Yale Zhang

**Affiliations:** 1 Department of Pediatrics，Affiliated Hospital of Shaanxi University of Chinese Medicine，Xianyang，712000，China; 2 Faculty of Rehabilitation and Allied Health Sciences, Riphah International University, Islamabad, Pakistan; 3 International Center of Medical Sciences Research - ICMSR, Islamabad, Pakistan; 4 International Center of Medical Sciences Research - ICMSR, Austin, TX, USA; 5 International Center of Medical Sciences Research - ICMSR, Essex, United Kingdom; 6 Operational Research Center in Healthcare, Near East University, Nicosia/TRNC, 99138 Mersin 10, Turkey; 7 Korea University College of Health Science, Korea University, Seongbuk-gu, Seoul, South Korea; 8 Clinical and Biomedical Research Center (CBRC), Foundation University School of Health Sciences (FUSH), Foundation University Islamabad, PAKISTAN; 9 University of Sharjah, College of Health Sciences, Medical Diagnostic Imaging Department, Sharjah, United Arab Emirates; 10 University of Sharjah, Research Institute for Medical and Health Sciences, Sharjah, United Arab Emirates; 11 Department of Pediatric Cardiovascular and respiratory， Xianyang Rainbow Hospital，Xianyang，712000，China

**Keywords:** Chronic Bacterial Pneumonia, *Pseudomonas aeruginosa*, Juvenile Lung Infection, Inflammation, Oxidative Stress, DNA Damage, Oncogenesis, NF-κB, Myc, Kras, Pharmacological Intervention

## Abstract

**Background:**

Chronic pulmonary infections pose a significant health burden, with accumulating evidence suggesting their potential to trigger oncogenic transformation. However, the link between chronic bacterial pneumonia and early neoplastic changes remains poorly understood, particularly in juvenile lungs.

**Objectives:**

The present study investigates how repeated *Pseudomonas aeruginosa* infection induces inflammation, oxidative stress, DNA damage, and oncogenic signaling in juvenile mice, and explores potential pharmacological targets to prevent long-term oncogenic consequences.

**Methods:**

Juvenile BALB/c mice received intranasal challenges with *P. aeruginosa* on days 0, 5, and 10. Lung tissues were collected at baseline (day 0) and after the establishment of chronic infection (day 21) for all downstream analyses. Lung tissues were analyzed for inflammatory [factor-kappa B (NF-κB), cyclooxygenase-2 (COX-2), tumor necrosis factor-alpha (TNF-α)], oxidative [nuclear factor erythroid 2-related factor 2 (Nrf2), heme oxygenase-1 (HO-1)], and DNA damage (γH2AX) markers using Western blotting, quantitative real-time PCR (qPCR), and immunofluorescence microscopy. Cell viability was assessed using MTT assays, and wound healing capacity was evaluated through scratch assays. Oncogenic markers (Myc, Kras) were quantified by qPCR.

**Results:**

Chronic *P. aeruginosa* infection led to persistent upregulation of inflammatory proteins (NF-κB, COX-2, TNF-α) and oxidative stress markers (Nrf2, HO-1) in lung tissues on day 21 compared to day 0. Increased γH2AX expression indicated DNA damage, although no significant DNA fragmentation was detected, suggesting sublethal, localized damage. Functionally, chronic infection resulted in a 35% reduction in cell viability and significantly delayed wound healing (60% closure compared to 90% in controls). Importantly, infected tissues displayed a 2.8-fold increase in Myc and a 2.5-fold increase in Kras mRNA levels, indicating early oncogenic signaling.

**Conclusions:**

Chronic *P. aeruginosa* infection in juvenile mice induces a sustained inflammatory and oxidative response, leading to epithelial cell dysfunction and activation of oncogenic pathways. These findings highlight the need for early therapeutic intervention targeting inflammation and oxidative stress to mitigate malignant transformation risks associated with recurrent pediatric lung infections. Agents modulating NF-κB activity or enhancing antioxidant defenses, such as Nrf2 activators, may represent promising pharmacological strategies. Early intervention and monitoring of chronic lung infections in pediatric populations are essential to mitigate potential oncogenic risks.

## 1. Background

Chronic lung infections remain a critical concern in both pediatric and adult populations due to their persistent effects on pulmonary tissue architecture and function (1). Unlike acute infections, which are transient and self-limiting, chronic infections can provoke prolonged inflammation and tissue remodeling, resulting in long-term consequences (2). Particularly in juveniles, whose lungs are still undergoing developmental changes, such persistent insults can predispose them to irreversible damage, increasing vulnerability to subsequent respiratory diseases and even malignancy (3). The respiratory tract, constantly exposed to environmental pathogens and irritants, relies on a finely tuned immune system to mount a defense (4). However, recurrent infections can overwhelm these mechanisms, shifting the immune response from a protective acute phase to a deleterious chronic state (4). Chronic lung infections, especially those caused by *Pseudomonas aeruginosa* or *Haemophilus influenzae*, are known to initiate persistent activation of immune cells, including neutrophils and macrophages, which continuously release pro-inflammatory cytokines like tumor necrosis factor-alpha (TNF-α), interleukin-6 (IL-6), and interleukin-1β (IL-1β) (5, 6). This phenomenon is particularly evident in conditions such as cystic fibrosis or bronchiectasis. Central to this inflammatory process is the nuclear factor-kappa B (NF-κB) pathway, which orchestrates the transcription of various genes involved in inflammation and cell survival (7). Overactivation of this pathway leads to a pro-inflammatory microenvironment that not only damages tissue but also sets the stage for pathological signaling alterations (8). If left unregulated, this inflammatory state can shift from an adaptive response to one that promotes oncogenesis by facilitating genetic instability, suppressing apoptosis, and altering normal cellular differentiation (7, 8). Pharmacological inhibition of NF-κB has been proposed as a promising strategy to dampen chronic inflammation and reduce oncogenic risk.

One of the direct consequences of chronic inflammation is the overproduction of reactive oxygen species (ROS) and reactive nitrogen species (RNS), collectively contributing to oxidative stress (9). Neutrophils, in particular, generate large amounts of ROS during the respiratory burst aimed at destroying pathogens. However, sustained ROS release can damage lipids, proteins, and nucleic acids within host cells, thereby impairing cellular functions and compromising genomic integrity (10). In the lung, oxidative stress has been implicated in the pathogenesis of various chronic conditions, including chronic obstructive pulmonary disease (COPD), idiopathic pulmonary fibrosis (IPF), and lung cancer (11). The excessive generation of ROS leads to the oxidation of guanine bases in DNA, producing mutagenic lesions such as 8-oxo-2'-deoxyguanosine (8-oxo-dG), which can cause point mutations if not efficiently repaired. Moreover, oxidative stress disrupts mitochondrial integrity, amplifying cellular dysfunction and initiating apoptotic or necrotic pathways (12). To counteract ROS-induced damage, cells activate the nuclear factor erythroid 2-related factor 2 (Nrf2) pathway, which upregulates antioxidant defense genes, including heme oxygenase-1 (HO-1) and glutamate-cysteine ligase catalytic (GCLC) subunit (13). However, in the context of chronic infection, the antioxidant response may be insufficient to counterbalance the overwhelming ROS burden, leading to cumulative damage over time. Therapeutically, Nrf2 activators are under investigation for their potential to restore redox balance in chronic pulmonary diseases. The sustained presence of inflammatory and oxidative mediators creates a microenvironment that favors carcinogenesis. Chronic exposure to cytokines and ROS can lead to the activation of proto-oncogenes (e.g., Myc, Kras) and the inactivation of tumor suppressor genes (e.g., TP53), initiating the early steps of transformation (14). DNA damage responses become chronically engaged, as evidenced by the accumulation of markers such as γH2AX, indicating double-strand breaks. If repair systems become faulty or overwhelmed, mutations may persist and propagate through successive cell divisions (15).

Additionally, inflammation-associated pathways, including cyclooxygenase-2 (COX-2) and inducible nitric oxide synthase (iNOS), produce metabolites that further exacerbate DNA damage or promote cell proliferation and angiogenesis (16). For instance, COX-2 overexpression has been linked with poor prognosis in non-small cell lung carcinoma (NSCLC), highlighting its dual role in inflammation and cancer development (17). This highlights COX-2 inhibitors, such as celecoxib, as potential chemopreventive agents in chronic lung inflammation models. In juvenile models, the susceptibility to these effects may be exacerbated due to the immaturity of immune and antioxidant systems. Studies have shown that young animals exposed to inflammatory stimuli exhibit heightened oxidative damage and impaired resolution capacity compared to adults, suggesting a developmental vulnerability that can predispose them to long-term lung pathologies (18, 19). Children and adolescents exposed to chronic infections — whether due to genetic conditions, environmental exposures, or immune deficiencies — are particularly at risk. The developing lung is highly dynamic, involving extensive cell proliferation, differentiation, and alveolarization. Any interference with these processes, particularly through inflammatory or oxidative insults, can result in permanent alterations in lung architecture and cellular programming (20). Despite this known vulnerability, few studies have addressed pharmacological strategies to mitigate infection-driven oncogenic transformation during early lung development. There is a critical need to explore therapeutic interventions that can disrupt the cascade from chronic infection to DNA damage and proto-oncogene activation.

## 2. Objectives

Given the complex interplay between persistent infection, inflammation, oxidative stress, and early oncogenic signaling, this study aims to investigate the mechanistic link between chronic bacterial pneumonia and pre-neoplastic changes in juvenile lung tissue. We specifically employed a juvenile mouse model of recurrent *P. aeruginosa* infection to characterize inflammatory markers, oxidative stress responses, DNA damage signatures, cell viability, tissue regeneration, and the expression of key oncogenes. Our findings reveal that chronic pulmonary infection leads to sustained NF-κB and COX-2 activation, elevated pro-inflammatory cytokines, increased oxidative stress marked by Nrf2 and HO-1 upregulation, and evidence of DNA damage through γH2AX expression. Additionally, infected tissues exhibited impaired wound healing, reduced cell viability, and early activation of Myc and Kras, suggesting the emergence of a pro-oncogenic lung microenvironment. These insights underscore the potential utility of anti-inflammatory and antioxidant pharmacotherapies in mitigating the long-term oncogenic consequences of chronic lung infections in pediatric populations.

## 3. Methods

### 3.1. Animal Model and Experimental Design

#### 3.1.1. Animal Husbandry

Juvenile BALB/c mice (4 - 6 weeks old, both sexes) were obtained from a certified animal facility and housed in pathogen-free conditions at 22 ± 2°C with 12-hour light/dark cycles. Animals were maintained on a standard chow diet and had ad libitum access to water. All experimental protocols were approved by the Institutional Animal Care and Use Committee (IACUC), in accordance with national guidelines for the care and use of laboratory animals.

### 3.2. Chronic Lung Infection Induction

Chronic bacterial pneumonia was induced using *P. aeruginosa* (clinical isolate, strain PAO1). Mice were anesthetized with 2% isoflurane and intranasally instilled with 1 × 10^7^ colony-forming units (CFUs) of *P. aeruginosa* suspended in 50 μL sterile phosphate-buffered saline (PBS). The instillation was repeated on days 5 and 10, yielding a total of three doses over 15 days, to simulate recurrent infection and promote chronicity. A naive baseline group (day 0) was euthanized immediately before the first instillation to provide untouched reference tissue, while an age-matched sham-infected group received sterile PBS on the same three-day schedule and served as the “PBS” control in all figures. To assess the long-term effects of chronic infection and allow for the immune response to stabilize, mice were sacrificed on day 21 post-initial infection. This approach was chosen to capture the persistent inflammatory and oxidative stress responses that may develop after the acute infection phase has subsided. Each group consisted of at least 5 animals (n = 5), ensuring adequate power for statistical comparison.

### 3.3. Tissue Collection and Processing

On day 21 post-initial infection (6 days after the final bacterial challenge), mice were euthanized by CO_2_ asphyxiation. This timeline was selected to ensure that the chronic immune responses and potential early oncogenic changes were fully established. Lungs were excised, washed in ice-cold PBS, and divided for downstream applications:

- Right lung lobes were snap-frozen in liquid nitrogen for protein and RNA analysis.

- Left lung lobes were fixed in 10% neutral-buffered formalin for histology and immunofluorescence studies.

Tissue processing was performed under sterile conditions to avoid secondary contamination.

### 3.4. Western Blotting

Lung tissue lysates were centrifuged at 14,000 × g for 15 minutes at 4°C. Supernatants were quantified for protein content using the BCA assay. Equal amounts (30 µg) of total protein were resolved by SDS-PAGE and transferred to PVDF membranes. Membranes were blocked in 5% non-fat milk for 1 hour and incubated overnight at 4°C with primary antibodies: (1) Anti-NF-κB (p65); (2) anti-COX-2; (3) anti-TNF-α; (4) anti-Nrf2; (5) anti-HO-1; (6) anti-γH2AX; (7) anti-GAPDH (loading control). Following incubation with HRP-conjugated secondary antibodies, bands were visualized using chemiluminescence (ECL kit) and quantified using ImageJ software. Protein expression levels were normalized to GAPDH.

### 3.5. Quantitative Real-Time PCR

Total RNA was extracted using TRIzol reagent according to the manufacturer’s protocol. RNA purity and concentration were measured using a NanoDrop spectrophotometer. The cDNA was synthesized from 1 µg of total RNA using a reverse transcription kit. Quantitative real-time PCR (qPCR) was performed with SYBR Green Master Mix using gene-specific primers for:

Primer sequences: The oligonucleotides (5′ → 3′) used were:

- Il6: F-ACTCACCTCTTCAGAACGAATTG, R-CCATCTTTGGAAGGTTCAGGTTG

- Tnfa: F-AGCCGATGGGTTGTACCTTG, R-GTGGGTGAGGAGCACGTAGT

- COX-2: F-CACTACATCCTGACCCACTT, R-ATGCTCCTGCTTGAGTATGT

- Myc: F-GCTGCTTAGACGCTGGATTT, R-TGTTGCTGATCTCCGTTTCC

- Kras: F-TGGTAGTTGGAGCTGGTGTC, R-GCACAAAGAAAGCCCTCCCC

- Gapdh: F-AAGGTCGGTGTGAACGGATTTG, R-TGTAGACCATGTAGTTGAGGTCA

Reactions were run in triplicate on a QuantStudio real-time PCR system. The relative gene expression was calculated using the 2^-ΔΔCt^ method, with results expressed as fold changes relative to control samples.

### 3.6. Immunofluorescence Microscopy

Primary lung epithelial cells were isolated from mouse lung tissue using enzymatic digestion and plated on glass coverslips. Cells were cultured for 24 hours, washed with PBS, and fixed with 4% paraformaldehyde. After permeabilization with 0.2% Triton X-100, cells were blocked with 5% BSA and incubated overnight at 4°C with anti-γH2AX and anti-NF-κB antibodies. Alexa Fluor-conjugated secondary antibodies were used for detection. Nuclei were counterstained with DAPI. Coverslips were mounted using antifade reagent, and images were acquired using a confocal microscope (Zeiss LSM series). Fluorescence intensity and localization were analyzed using Fiji (ImageJ).

### 3.7. DNA Fragmentation Assay

Genomic DNA was extracted from lung tissues using a phenol-chloroform extraction method. DNA integrity was assessed by agarose gel electrophoresis (1.5% agarose, ethidium bromide-stained). Samples were visualized under UV light to detect fragmentation patterns. Intact high molecular weight DNA was indicative of minimal apoptosis.

### 3.8. Cell Viability (MTT Assay)

Primary lung epithelial cells were isolated and cultured from both infected and control mice. Cells were seeded in 96-well plates (1 × 10^4^ cells/well) and incubated with 0.5 mg/mL MTT for 4 hours. Formazan crystals were solubilized with DMSO, and absorbance was measured at 570 nm using a microplate reader. Viability was calculated as a percentage of the control group.

### 3.9. In Vitro Wound Healing Assay

Confluent monolayers of lung epithelial cells were mechanically scratched using a sterile pipette tip to create a uniform wound. Cells were washed to remove debris and cultured in serum-free medium. Images were captured at 0, 12, and 24 hours using a phase-contrast microscope. Wound closure was quantified by measuring the remaining gap area using ImageJ software. Wound width was standardized across replicates, and three random fields per sample were analyzed.

### 3.10. Statistical Analysis

 All data are presented as mean ± standard error of the mean (SEM). Statistical significance was determined using unpaired two-tailed Student’s *t*-tests for comparisons between two groups. For multiple group comparisons, one-way ANOVA followed by Tukey’s post-hoc test was used. A P-value of less than 0.05 was considered statistically significant. GraphPad Prism (version 9.0) was used for all analyses and data visualization. All experiments were performed in at least three biological replicates unless otherwise stated.

## 4. Results

### 4.1. Chronic Infection Induces Persistent Inflammation

**Figure 1.**
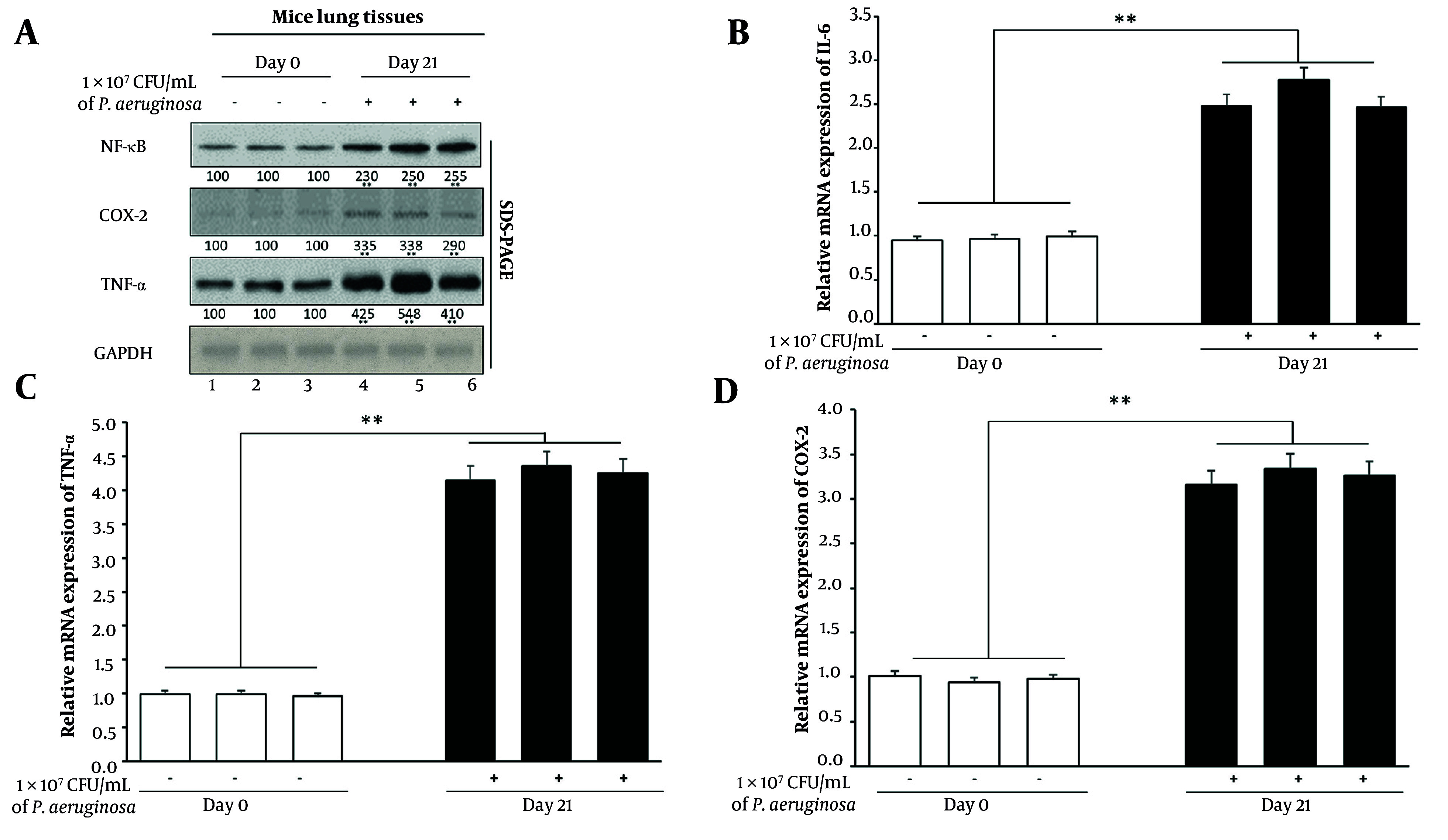
Chronic *Pseudomonas aeruginosa* infection promotes inflammatory signaling in juvenile mouse lungs. A, Western blot analysis of lung homogenates from naive (day 0) and chronically infected (day 21) mice. Protein expression levels of factor-kappa B (NF-κB), cyclooxygenase-2 (COX-2), and tumor necrosis factor-alpha (TNF-α) were evaluated. GAPDH served as a loading control. Densitometric quantification from three independent experiments is shown below the blots. B - D, quantitative real-time PCR (qPCR) analysis of inflammatory gene expression [interleukin-6 (IL-6) (B), tumor necrosis factor-alpha (TNF-α) (C), and cyclooxygenase-2 (COX-2) (D)] using RNA extracted from lungs harvested on day 0 and day 21. Data were normalized to GAPDH and represent mean ± standard error of the mean (SEM) from three biological replicates (** P < 0.001).

To determine whether chronic bacterial pneumonia triggers a sustained inflammatory response in juvenile lung tissue, we evaluated the expression of key inflammatory mediators at both the protein and mRNA levels (Figure 1). Lung tissues were collected at two time points: Day 0 (pre-infection) and day 21 (chronic stage, post-infection). Western blot analysis demonstrated a significant upregulation of pro-inflammatory proteins in lung tissues harvested from chronically infected mice. Specifically, expression levels of NF-κB-light-chain-enhancer of activated B cell, COX-2, and TNF-α were markedly elevated compared to uninfected controls (Figure 1A). Densitometric analysis from three independent biological replicates confirmed a 2.5-fold increase in NF-κB, a 3.1-fold increase in COX-2, and a 4.0-fold increase in TNF-α, normalized to GAPDH (P < 0.01) (Figure 1A). These molecules play central roles in inflammatory signaling and are commonly associated with chronic immune activation.

To validate these protein-level changes at the transcriptional level, qPCR was performed on RNA extracted from day 0 and day 21 lung tissues. Gene expression analysis revealed significantly increased mRNA levels of Il6, Tnfa, and COX-2 in infected lung tissues relative to controls (P < 0.01) (Figure 1B). The coordinated upregulation of both transcript and protein levels underscores the sustained activation of inflammatory pathways in response to chronic infection.

Persistent elevation of NF-κB and its downstream effectors on day 21 suggests ongoing inflammatory signaling that may contribute to structural lung remodeling and immune cell recruitment. Given the established role of chronic inflammation in tumor initiation, these findings indicate that recurrent pulmonary infection in juvenile mice fosters a pro-inflammatory microenvironment that could serve as a precursor to oncogenic transformation.

### 4.2. Chronic Infection Promotes Oxidative Stress and DNA Damage

**Figure 2.**
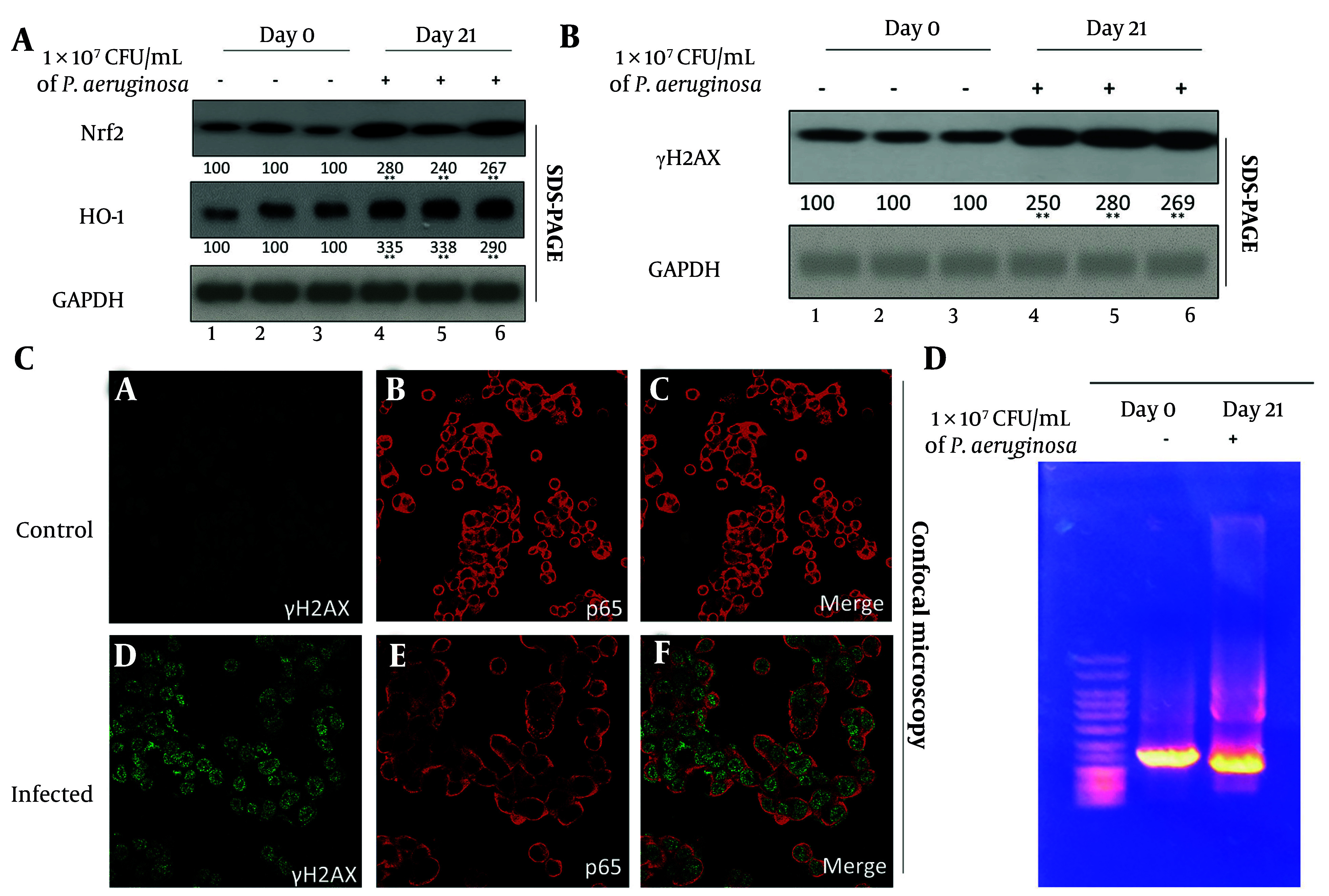
Chronic pulmonary infection induces oxidative stress and focal DNA damage in juvenile mouse lungs. A, Western blot analysis of antioxidant pathway components nuclear factor erythroid 2-related factor 2 (Nrf2) and heme oxygenase-1 (HO-1) in lung tissues from day 0 (control) and day 21 (infected) mice. GAPDH was used as a loading control. Densitometric quantification are shown below the blots from three independent blots. B , Western blot detection of γH2AX as a marker of DNA double-strand breaks in lung homogenates from the same samples. Densitometric analysis are shown below the blots. C, confocal immunofluorescence microscopy of cultured primary lung epithelial cells stained for γH2AX (green) and p65 (red) to localize DNA damage within epithelial nuclei. Representative images from control (day 0) and infected (day 21) mice are shown. D, agarose gel electrophoresis of genomic DNA extracted from control and infected lungs to assess integrity and potential fragmentation patterns. Each lane represents an individual sample from the indicated group.

Oxidative stress is a well-documented consequence of chronic inflammation and a major contributor to genomic instability. To investigate this phenomenon, we analyzed the expression of Nrf2 and its downstream effector, HO-1, both of which are crucial components of the antioxidant defense system. Western blot analysis showed a 2.8-fold increase in Nrf2 and a 3.5-fold increase in HO-1 in the lungs of infected mice relative to controls (P < 0.001) (Figure 2A). These data suggest that cells within the infected lung tissue are responding to heightened oxidative stress by upregulating protective antioxidant pathways.

Despite this upregulation of protective mechanisms, signs of genotoxic stress were evident. γH2AX — a phosphorylated histone variant and well-established marker of DNA double-strand breaks — was significantly increased in infected lung tissues (P < 0.001), as shown by Western blot (Figure 2B). The accumulation of γH2AX indicates that the oxidative burden exceeded the reparative capacity of the tissue, leading to DNA damage. To further localize this damage, confocal immunofluorescence microscopy was employed. Cultured primary lung epithelial cells isolated from *P. aeruginosa*-infected mice exhibited distinct γH2AX-positive nuclear foci, indicative of active DNA damage response pathways (Figure 2C, lower panel). In contrast, control tissues displayed minimal or diffuse γH2AX staining. The nuclear localization of γH2AX foci confirms the site-specific impact of oxidative stress on genomic DNA and supports the presence of early-stage DNA damage, rather than non-specific injury or cell death.

Interestingly, despite the upregulation of γH2AX, agarose gel electrophoresis of genomic DNA from both infected and control tissues showed intact, high molecular weight bands; however, the day 21 samples also exhibited a faint "tail" or slight smear, suggestive of mild DNA damage (Figure 2D). Given the significant increase in γH2AX expression, it is plausible that the observed DNA damage primarily consists of localized double-strand breaks that are not widespread enough to result in large-scale fragmentation detectable by gel electrophoresis. This pattern suggests that chronic oxidative stress induced sublethal, focal DNA damage rather than extensive apoptosis or necrosis. Such sublethal genotoxic stress is significant because it allows for the survival of damaged cells, which may accumulate mutations, increasing the risk of malignant transformation over time.

### 4.3. Chronic Infection Reduces Cell Viability and Delays Wound Healing

**Figure 3.**
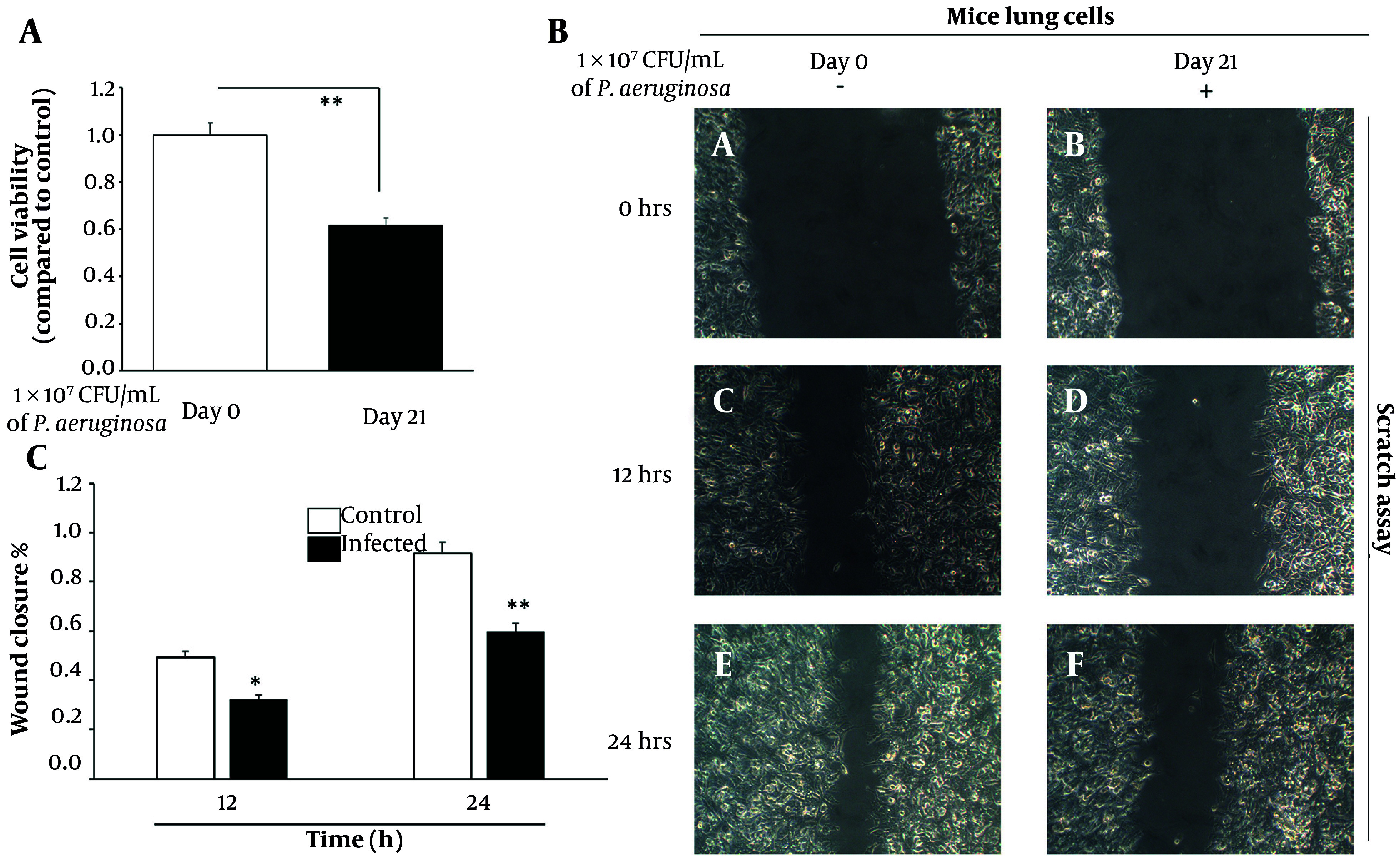
Chronic *Pseudomonas aeruginosa* infection impairs lung epithelial cell viability and delays wound healing. A, cell viability assessed by MTT assay in primary lung epithelial cells isolated from day 0 (control) and day 21 (infected) mouse lungs. Absorbance values were normalized to day 0 controls. Data represent mean ± standard error of the mean (SEM) from three independent experiments. B, representative phase-contrast images from scratch wound healing assays at 0 h, 12 h, and 24 h post-scratch, comparing control and infected lung epithelial cells. C, quantification of wound closure (%) at 12 h and 24 h using bar graphs. Data represent mean ± SD from three biological replicates. Initial scratch width was set as 100%, and remaining wound area was measured at each time point (* P < 0.01 and ** P < 0.001).

The functional consequences of chronic inflammation and oxidative stress were next assessed by evaluating cell viability and regenerative capacity. The MTT assay demonstrated a significant 35% decrease in metabolic activity of lung epithelial cells isolated from infected tissues on day 21, relative to day 0 controls (P < 0.001) (Figure 3A). This decline in viability likely reflects the cumulative effects of cellular stress, including DNA damage, cytokine overload, and oxidative burden, which collectively contribute to reduced proliferation and possibly increased apoptosis.

To evaluate tissue regenerative function, wound healing assays were performed using scratch migration techniques. Control lung epithelial cells exhibited efficient migration, achieving approximately 50% wound closure within 12 hours and nearly 90% closure by 24 hours. In contrast, cells derived from chronically infected lungs closed only 30% of the wound area at 12 hours and a maximum of 60% at 24 hours (P < 0.01 and P < 0.001, respectively) (Figure 3B). This pronounced delay in wound closure is indicative of impaired cellular motility and proliferative capacity, both of which are essential for tissue regeneration and barrier restoration following injury. These results were quantitatively confirmed using a bar graph representation of wound closure percentages at 12 h and 24 h (Figure 3C), clearly illustrating the compromised migration capacity of infected cells compared to controls. Chronic inflammation, therefore, not only compromises cell survival but also hampers functional recovery of the lung epithelium, further predisposing the tissue to pathological remodeling and oncogenic transformation.

### 4.4. Chronic Pneumonia Induces Oncogenic Signaling Pathways

**Figure 4.**
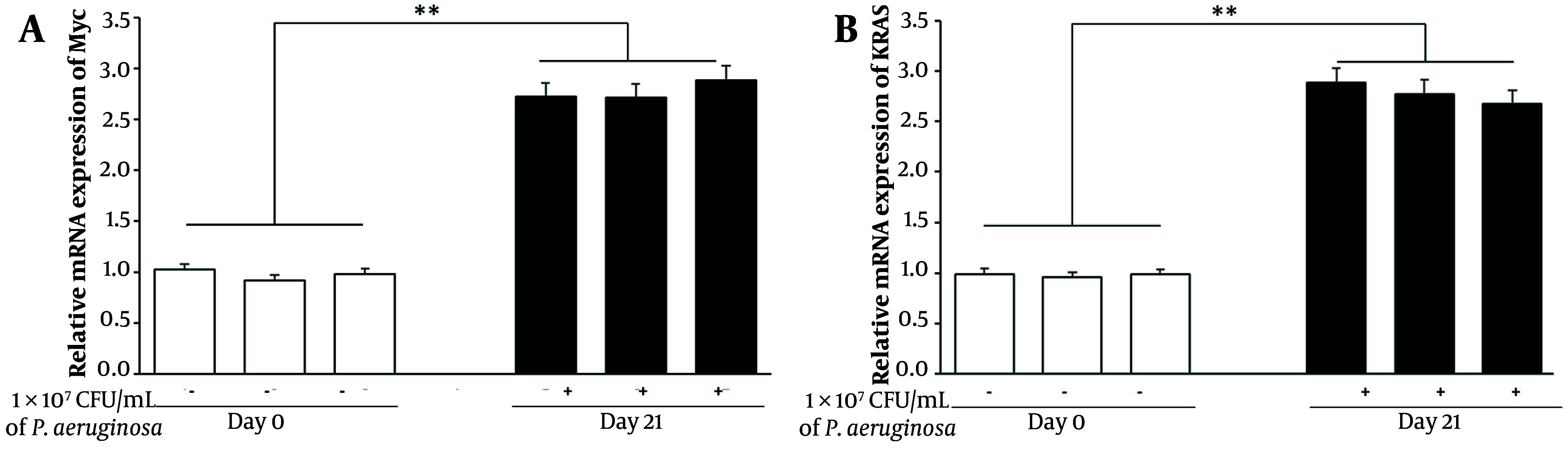
Chronic *Pseudomonas aeruginosa* infection induces early oncogenic signaling in juvenile mouse lungs. Quantitative real-time PCR (qPCR) analysis of Myc and Kras gene expression in lung tissues collected from control (day 0) and chronically infected (day 21) mice. RNA was isolated, reverse-transcribed, and analyzed using SYBR Green-based qPCR. Gene expression levels were normalized to GAPDH and presented as fold changes relative to day 0 controls. Data represent mean ± standard error of the mean (SEM) from three biological replicates per group (** P < 0.001).

To assess the potential for oncogenic transformation, we evaluated the expression of oncogenic markers Myc and Kras by qPCR. Analysis showed a 2.8-fold increase in Myc and a 2.5-fold increase in Kras mRNA levels in lung tissues from infected mice on day 21 relative to day 0 (P < 0.001) (Figure 4). Elevated expression of these proto-oncogenes in the context of chronic lung infection suggests a shift toward a pre-malignant state within the epithelial compartment.

Interestingly, while both Myc and Kras are associated with tumor initiation, the observed fold increases are somewhat modest compared to previous literature on inflammation-driven oncogenesis. This could indicate that the chronic bacterial infection model primarily induces early transformation markers rather than fully activating oncogenic signaling. Further validation through longer time-point studies may be necessary to observe full malignant progression.

**Figure 5.**
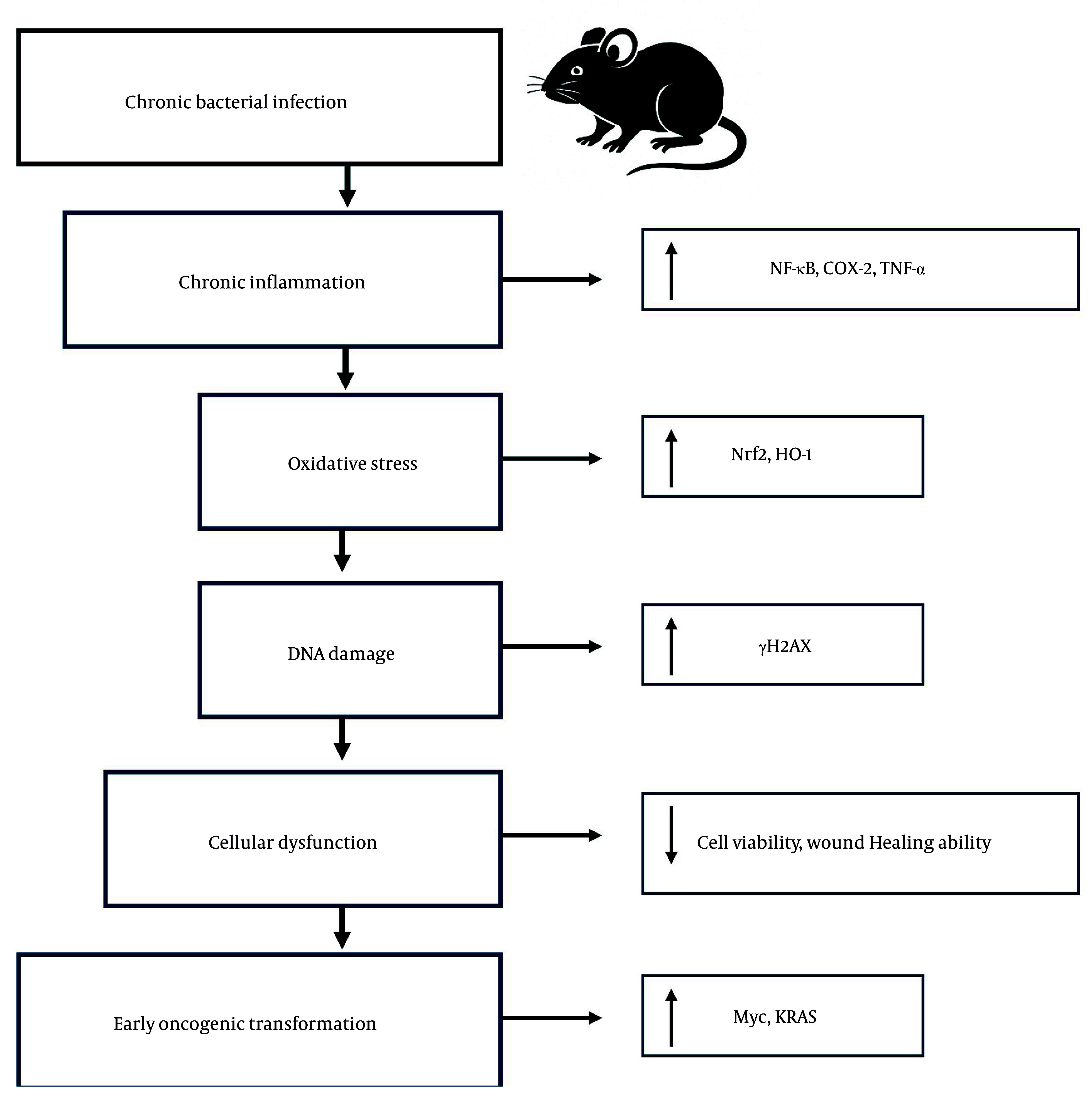
Proposed mechanistic model linking chronic bacterial pneumonia to early oncogenic transformation in juvenile lungs. Chronic *Pseudomonas aeruginosa* infection in juvenile mice induces a persistent inflammatory response characterized by elevated factor-kappa B (NF-κB), tumor necrosis factor-alpha (TNF-α), and cyclooxygenase-2 (COX-2) signaling. This inflammation promotes oxidative stress, triggering activation of the nuclear factor erythroid 2-related factor 2 (Nrf2)-heme-oxygenase-1 (HO-1) antioxidant pathway. Despite this response, sublethal DNA damage accumulates, as evidenced by increased γH2AX. These molecular events lead to reduced epithelial cell viability, impaired wound healing, and upregulation of proto-oncogenes such as Myc and Kras. Together, these alterations converge to create a pro-tumorigenic microenvironment. Arrows indicate the direction of effect; red pathways denote damage or dysfunction, while green elements represent protective responses.

Taken together, the experimental data demonstrate that chronic bacterial pneumonia in juvenile mice elicits a multifaceted pathological response. Key hallmarks include sustained pro-inflammatory signaling (NF-κB, TNF-α, COX-2), increased oxidative stress (Nrf2, HO-1), early DNA damage (γH2AX), diminished epithelial cell viability, impaired wound healing ability, and activation of oncogenic programs (Myc, Kras) (Figure 5). The interplay of these processes suggests that chronic pulmonary infection does not merely damage tissue in the short term but may also serve as a predisposing factor for malignancy in the long term. These findings underscore the critical need for early diagnosis, effective management of chronic lung infections, and long-term monitoring of affected individuals to mitigate potential oncogenic progression.

## 5. Discussion

Chronic infections and cancer are two of the most formidable health burdens worldwide, each driving long-term morbidity and mortality through distinct yet sometimes interconnected pathways (21-24). While chronic infections like hepatitis B and C viruses, Epstein-Barr virus, and *Helicobacter pylori* are well-established carcinogenic risks in the liver and stomach, respectively, the potential for chronic respiratory infections — particularly bacterial pneumonia — to initiate oncogenic transformation remains underexplored. This study provides compelling evidence that chronic bacterial pneumonia in juvenile mice, modeled via *P. aeruginosa* infection, can initiate a cascade of pathological events culminating in a lung microenvironment conducive to early neoplastic transformation. To our knowledge, this is among the first preclinical studies directly linking repeated pediatric lung infection with inflammation-driven proto-oncogene activation in a controlled experimental model.

A central finding of this work is the persistent activation of inflammatory pathways following repeated pulmonary infection. Upregulation of NF-κB, COX-2, and TNF-α at both the transcriptional and protein levels on day 21 highlights the extent of inflammation sustained after the infection protocol. This mirrors prior studies showing that unresolved inflammation is a key contributor to both acute lung injury and the evolution of chronic lung disease (25). Moreover, the magnitude of upregulation — up to 4.0-fold in TNF-α — demonstrates the strength of the inflammatory stimulus. The NF-κB, in particular, is a master regulator of inflammation and is frequently found constitutively active in many human cancers due to its role in promoting cell survival, proliferation, and cytokine production (26). These findings suggest that NF-κB inhibitors or COX-2-targeted agents, such as celecoxib, may hold therapeutic potential in preventing inflammation-associated lung damage in high-risk pediatric populations.

Concomitant with inflammatory signaling, we observed significant oxidative stress within infected lung tissues. The Nrf2 and HO-1, critical components of the antioxidant defense response, were markedly elevated, suggesting an adaptive but potentially insufficient countermeasure to ongoing ROS production. *Pseudomonas aeruginosa* is known to generate high levels of ROS through virulence factors like pyocyanin and rhamnolipids, which disrupt epithelial integrity and provoke sustained neutrophilic infiltration. Persistent oxidative stress is a recognized promoter of carcinogenesis, as it facilitates DNA lesions, lipid peroxidation, and mutagenesis (27, 28). In this context, activation of the Nrf2 pathway, although evident, may not fully mitigate ROS damage, warranting investigation into adjunct pharmacological antioxidant therapies such as sulforaphane, N-acetylcysteine, or bardoxolone methyl.

Evidence of genotoxic stress was further confirmed by the elevated expression and nuclear localization of γH2AX — a marker of DNA double-strand breaks. While agarose gel electrophoresis did not reveal large-scale DNA fragmentation, the presence of γH2AX foci implies early, sublethal DNA damage capable of contributing to genomic instability. The absence of significant DNA fragmentation suggests that the oxidative damage is focal and does not reach the threshold required to trigger widespread apoptosis. This pattern is consistent with sublethal stress that may allow survival of damaged cells, potentially leading to the accumulation of mutations over time (29). Importantly, such damage in proliferating juvenile lung epithelial cells may persist into adulthood, establishing long-term oncogenic risk.

Functionally, this molecular damage translated to reduced cell viability and impaired wound healing. A 35% reduction in MTT assay activity and a 30 - 40% delay in wound closure on day 21 highlight the physiological toll of chronic infection on lung tissue regeneration. These findings suggest that beyond promoting inflammation, *P. aeruginosa* may interfere with epithelial renewal, further compromising barrier integrity and potentiating long-term structural damage. This is consistent with studies in chronic COPD, where oxidative stress and impaired repair mechanisms are hallmarks of disease progression (30). Therapeutic interventions that promote epithelial regeneration, such as growth factor analogs or stem-cell-derived exosomes, may also be worth exploring in future models.

Crucially, our data show that chronic infection activates oncogenic signaling pathways, with significant upregulation of Myc and Kras — two proto-oncogenes commonly mutated or overexpressed in lung cancer. Their increased expression on day 21 suggests that repeated pulmonary infection may drive the tissue toward a pre-malignant state through inflammatory and oxidative mechanisms. Previous research has implicated Myc and Kras as central players in inflammation-associated tumorigenesis, capable of reprogramming cellular metabolism and promoting proliferation in damaged tissues (31). Our findings support the concept that chronic infection acts as both an initiating and promoting factor in early lung oncogenesis.

Taken together, these findings support a multi-hit model of transformation in which chronic infection acts as both initiator and promoter of early oncogenic events. This is particularly concerning in juvenile lungs, where developmental processes are still ongoing and may be permanently altered by persistent inflammatory and oxidative cues. The juvenile period is critical for lung alveolarization and immune programming; disruption during this window may predispose individuals to chronic lung disease and cancer in adulthood (32). Therefore, surveillance and early intervention in children with repeated lower respiratory tract infections could play a preventive role in long-term respiratory malignancy risk.

### 5.1. Conclusions

In conclusion, our study provides novel mechanistic insights into how chronic *P. aeruginosa* infection can reshape the juvenile lung microenvironment through inflammation, oxidative stress, and activation of oncogenic pathways. These results underscore the urgency of early diagnosis and management of recurrent pulmonary infections in children — not only to preserve immediate lung function but also to reduce the long-term risk of malignancy. From a translational perspective, these findings advocate for the investigation of combined anti-inflammatory and antioxidant therapies as a pharmacological strategy to mitigate infection-driven carcinogenic risk. Future studies should investigate the reversibility of these changes with anti-inflammatory or antioxidant therapies and assess the relevance of these findings in human pediatric populations.

## Data Availability

The data presented in this study are uploaded during submission as a supplementary file and are openly available for readers upon request.
